# Meckel’s Diverticulum as an Unexpected Cause of Acute Intestinal Obstruction in an Adult

**DOI:** 10.7759/cureus.108652

**Published:** 2026-05-11

**Authors:** Anass Barchid, Issam Yazough, Hamza Yousri, Younes Aggouri, Said Aitlaalim

**Affiliations:** 1 General Surgery, Faculty of Medicine and Pharmacy, Mohammed VI University Hospital, Abdelmalek Essaâdi University, Tangier, MAR

**Keywords:** acute intestinal obstruction, bowel pathology, intestinal obstruction, meckel's diverticulum, secondary volvulus

## Abstract

Meckel’s diverticulum is a congenital anomaly that often remains silent and is found incidentally. In adults, it may present with complications such as bowel obstruction or inflammation, sometimes precipitated by intussusception or volvulus. Imaging can raise suspicion of related complications, but confirmation frequently occurs at surgery. Operative management is recommended when complications are present, whereas prophylactic removal of incidentally discovered lesions remains a subject of debate.

We present a case of a 56-year-old male with no significant medical history who presented with abdominal pain, cessation of bowel movements and gas, and episodes of vomiting. Initial examination revealed a stable patient with mild abdominal tenderness. Abdominal X-ray and CT scan demonstrated signs of mechanical small bowel obstruction. The obstruction was caused by a Meckel’s diverticulum, identified during surgery and treated with resection of the affected small intestinal segment. The patient had an uneventful recovery. This case highlights the importance of considering Meckel’s diverticulum in the differential diagnosis of small bowel obstruction, even in adults. This case report aimed to emphasize that Meckel’s diverticulum, although usually asymptomatic and rarely suspected in adults, should be considered in the differential diagnosis of acute small bowel obstruction, especially when no common obstructive cause is identified on imaging.

## Introduction

Meckel’s diverticulum is the most common congenital anomaly of the gastrointestinal tract [1]. It arises from the incomplete obliteration of the vitelline duct, which connects the primitive midgut to the yolk sac during early fetal development [2]. Anatomically, Meckel’s diverticulum is classified as a true diverticulum, comprising all layers of the intestinal wall, and is typically located on the antimesenteric border of the ileum [3]. In most cases, it remains clinically silent [4]. However, when symptomatic, it may present with complications such as gastrointestinal bleeding, intestinal obstruction, or inflammation [3].

The clinical presentation varies by age as follows: in children, Meckel’s diverticulum often manifests as painless rectal bleeding or may lead to intussusception, whereas in adults, it more commonly presents with signs of obstruction or diverticulitis [5]. The potential morbidity associated with Meckel’s diverticulum is largely related to these complications [2].

## Case presentation

We present here a case of a 56-year-old male with no relevant medical history who presented with a three-day history of abdominal pain, followed by cessation of bowel movements and gas, and associated with episodes of alimentary vomiting. These symptoms prompted him to seek medical care at the emergency department.

On general examination, the patient was conscious and hemodynamically stable. His Glasgow Coma Scale (GCS) score was 15/15, blood pressure measured at 110/60 mmHg, heart rate was 97 beats per minute (bpm), oxygen saturation was 98% on room air, and body temperature was elevated at 38.3°C. Abdominal examination revealed a non-distended abdomen with generalized tenderness. Digital rectal examination showed the presence of traces of stool. The remainder of the physical examination was unremarkable.

**Figure 1:**
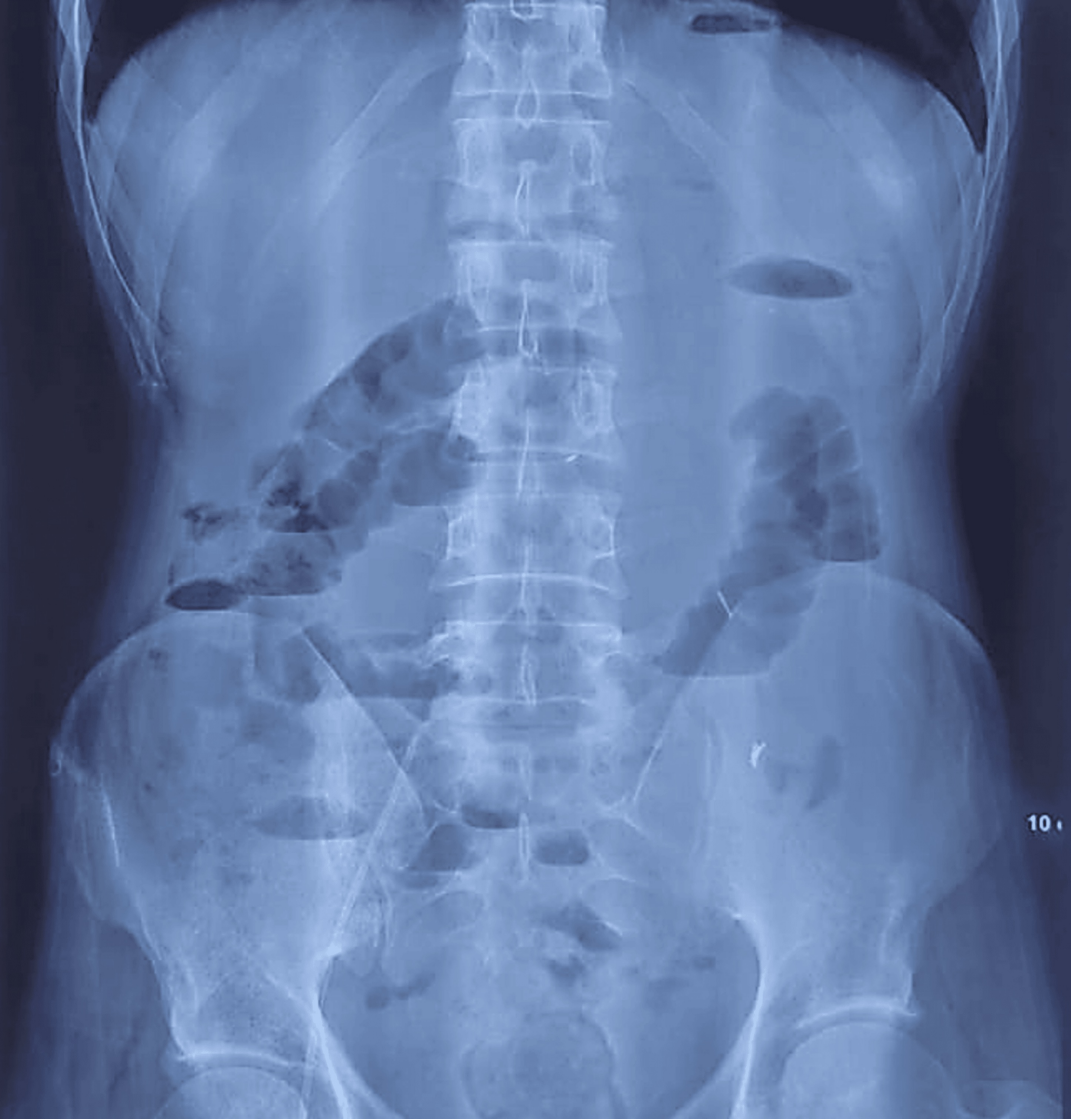
Abdominal X-ray showing multiple air-fluid levels suggestive of bowel obstruction.

The abdominal X-ray performed in the supine position revealed multiple air-fluid levels predominantly located in the small bowel, findings that are highly suggestive of a mechanical small bowel obstruction. No evidence of pneumoperitoneum, such as free subdiaphragmatic air, was detected (Figure 1).

**Figure 2:**
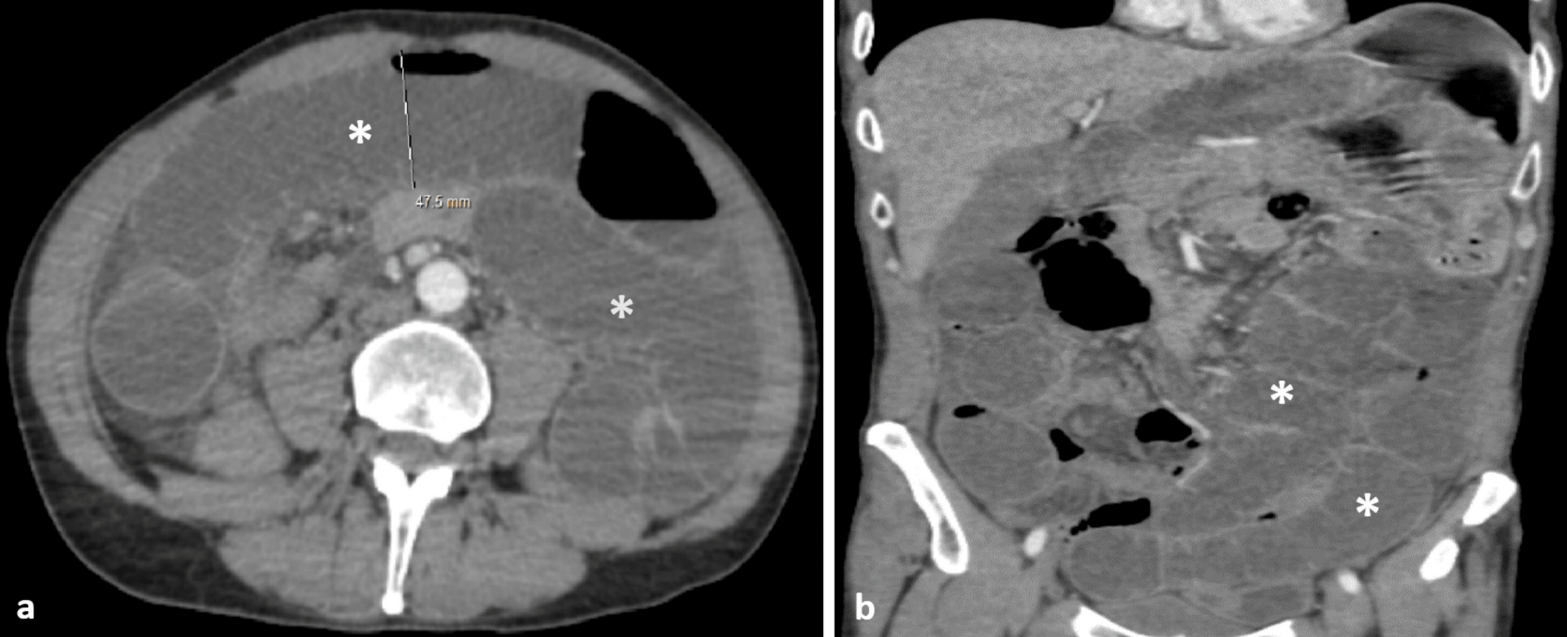
Abdominal CT scan showing markedly distended bowel loops (*) with a coffee-bean appearance, without visible Meckel’s diverticulum. (a) Transverse view and (b) coronal view.

Further evaluation with an abdominal CT scan with contrast confirmed the presence of a small bowel obstruction with identification of a clear transition point. The imaging showed dilated loops of small intestine upstream from the obstruction, with a maximum diameter of approximately 3.5 cm. Distally, the bowel segments appeared collapsed beyond the transition point. There were no radiological signs suggestive of bowel ischemia, including no parietal pneumatosis or portomesenteric venous gas. Additionally, no intra-abdominal collections or significant lymphadenopathy were noted. Importantly, no mass lesion or hernia was identified as the underlying cause of the obstruction (Figures 2a, 2b). Laboratory investigations revealed normal liver enzyme levels with aspartate aminotransferase (AST) at 14 U/L and alanine aminotransferase (ALT) at 19 U/L. Total bilirubin was 12 µmol/L, direct bilirubin was 4 µmol/L, and indirect bilirubin was 8 µmol/L. Alkaline phosphatase (ALP) and gamma-glutamyl transferase (GGT) levels were within normal ranges, at 78 U/L and 22 U/L, respectively. Other key laboratory findings are summarized in Table 1.

**Table 1:** Admission laboratory results.

Parameters	Result	Normal range
Hemoglobin (Hb)	16.5 g/dL	13-17 g/dL
White blood cells (WBC)	5,430/mm³	4,000-10,000/mm³
Neutrophils (PNN)	4,494/mm³	2,000-7,000/mm³
Platelets (Pq)	242,000/mm³	150,000-400,000/mm³
C-reactive protein (CRP)	29 mg/L	<6 mg/L
Lipase	11 U/L	13-60 U/L
Urea	0.73 g/L	0.15-0.45 g/L
Creatinine	13.9 mg/L	6-12 mg/L
Sodium (Na⁺)	139 mmol/L	135-145 mmol/L
Potassium (K⁺)	4.1 mmol/L	3.5-5.1 mmol/L
Aspartate aminotransferase (AST)	14 U/L	5-40 U/L
Alanine aminotransferase (ALT)	19 U/L	5-40 U/L
Total bilirubin	12 µmol/L	<17 µmol/L
Direct bilirubin	4 µmol/L	<5 µmol/L
Indirect bilirubin	8 µmol/L	<12 µmol/L
Alkaline phosphatase (ALP)	78 U/L	40-130 U/L
Gamma-glutamyl transferase (GGT)	22 U/L	10-50 U/L

Initial management included hospitalization with clinical monitoring and stabilization. A nasogastric tube was placed, and the patient received intravenous rehydration therapy. Analgesia was provided with acetaminophen and codeine, and antibiotic therapy was initiated with amoxicillin and clavulanic acid. Amoxicillin-clavulanic acid was selected to cover common community-acquired enteric Gram-negative and anaerobic bacteria. Additionally, an antispasmodic and an antiemetic were administered.

**Figure 3:**
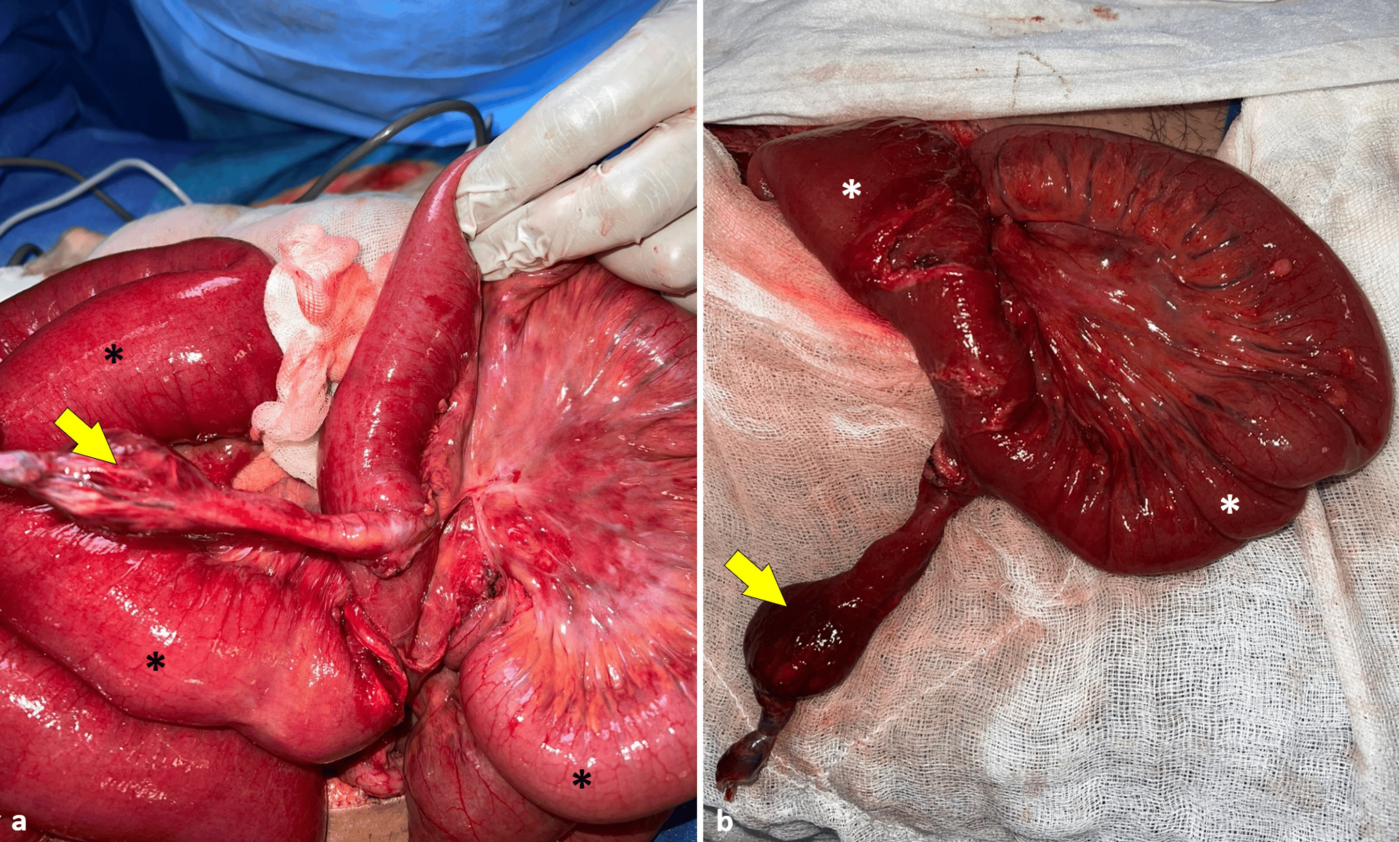
Intraoperative findings showing (a and b) the Meckel’s diverticulum (arrow) and the distended small-intestinal segments (*).

A midline laparotomy was performed under general anesthesia. Exploration revealed dilated small bowel loops upstream of an obstruction point caused by a Meckel’s diverticulum with surrounding inflammatory adhesions. The segment of small intestine containing the Meckel’s diverticulum was resected (Figures 3a, 3b). A side-to-side, hand-sewn anastomosis was fashioned and tested for integrity with no leakage observed. No other intra-abdominal abnormalities were noted.

The abdominal cavity was irrigated with warm saline. A drain was placed adjacent to the anastomosis. The abdominal wall was closed in layers. Postoperatively, the patient tolerated the procedure well, with stable vital signs and no complications. He was discharged on postoperative day three with instructions for wound care and a follow-up appointment scheduled.

## Discussion

Meckel’s diverticulum (MD) is a congenital anomaly that is typically discovered incidentally. It is the most common congenital gastrointestinal malformation, affecting up to 3% of the population [6]. The anomaly was first described by German anatomist Johann Friedrich Meckel [2]. MD arises from incomplete closure of the vitelline duct during the fifth week of embryonic development [1]. The vitelline duct serves as a connection between the developing intestines and the yolk sac during early development [7]. MD manifests as a true outpouching of the small intestine, typically located about 60 cm from the ileocecal valve [1].

MD is found in about 2% of the general population and is more frequently observed in male children [3,8]. Although MD is rarely diagnosed in adults, it should be considered in the differential diagnosis of acute abdominal pain and bowel obstruction [4]. The diverticulum is positioned along the antimesenteric border of the ileum, usually between 7 cm and 200 cm from the ileocecal valve [3].

In most cases, MD is asymptomatic and is often identified incidentally during surgery for unrelated conditions or during imaging studies [4,9]. The lifetime risk of complications is approximately 5%, with 40% of these occurring in children under 10 years of age [3]. In children, the most common complication is painless gastrointestinal bleeding, observed in 50% of cases. This bleeding typically results from ectopic gastric mucosa within the diverticulum but can also lead to more severe issues, such as acute intestinal obstruction [1]. In adults, the primary complications include bowel obstruction and diverticulitis [10]. Obstruction may be caused by various mechanisms, including intussusception, volvulus, abdominal hernia, or entrapment of the diverticulum in the mesentery [3,10].

Imaging studies often reveal air-fluid levels, indicating pathological fluid and gas accumulation, which is a characteristic finding on X-rays and CT scans of bowel obstruction [11]. X-rays are typically limited in diagnostic value but may reveal enteroliths or signs of bowel obstruction [2]. Ultrasound can detect mesodiverticular bands as hyperechoic lines, particularly in pediatric patients. CT scans are generally more accurate in identifying the cause of small bowel obstruction in adults, although the diverticulum may not always be visible in the absence of complications [1]. In cases of intestinal bleeding, angiography can identify a remnant omphalomesenteric artery [12]. MD is located on the antimesenteric side of the distal ileum, distinguishing it from alimentary duplications and other bowel diverticula [2].

MD is a true diverticulum composed of all the layers of the gastrointestinal tract as follows: mucosa, submucosa, muscular, and serosal layers. It often contains ectopic gastric mucosa, which can be identified during microscopic examination [3,9]. In approximately 50% of cases, the diverticulum contains ectopic tissue, most commonly gastric mucosa, which is linked to complications such as hemorrhage, inflammation, or perforation. Pancreatic tissue may also be present in some cases.

Surgical removal of MD, via diverticulectomy, wedge resection, or segmental resection, is the primary treatment, depending on the condition of the diverticulum and the proximal ileum [13]. Surgical removal is mandatory in cases where complications like torsion, obstruction, volvulus, or perforation occur [3]. Wedge or segmental resection is recommended when MD causes bowel obstruction. The question of prophylactic resection for incidental MD remains controversial, as no consensus guidelines advocate routine removal of asymptomatic MD. However, considering the potential complications and the relative ease of surgical resection, prophylactic resection might be justified when incidental MD is discovered [1,2]. Although MD is uncommon in adults, it should be considered in the differential diagnosis of bowel obstruction, particularly after ruling out more common causes such as postoperative adhesions and hernias [1].

## Conclusions

Meckel’s diverticulum, although often asymptomatic, can lead to significant complications such as bowel obstruction, even in adults. This case underscores the importance of maintaining a high index of suspicion for Meckel’s diverticulum when evaluating small bowel obstruction in adult patients, especially in the absence of prior abdominal surgeries or other common causes. Imaging studies, while helpful, may not always clearly identify the underlying cause, making surgical exploration essential for definitive diagnosis and treatment. Timely surgical intervention remains critical to prevent serious outcomes, and segmental small bowel resection offers an effective and curative approach. Given the potential for severe complications, the management of incidentally discovered Meckel’s diverticulum during surgery should be carefully considered on a case-by-case basis.
